# Hospital variation in admissions for low back pain following an emergency department presentation: a retrospective study

**DOI:** 10.1186/s12913-022-08134-8

**Published:** 2022-07-12

**Authors:** Giovanni Ferreira, Marina Lobo, Bethan Richards, Michael Dinh, Chris Maher

**Affiliations:** 1 Institute for Musculoskeletal Health, Sydney Local Health District, Sydney, Australia; 2 School of Public Health, Faculty of Medicine and Health, The University of Sydney, Sydney, NSW Australia; 3 Camperdown, Australia; 4 Center for Health Technology and Services Research (CINTESIS), Department of Community Medicine, Information and Health Decision Sciences (MEDCIDS), Faculty of Medicine, University of Porto, Porto, Portugal; 5 The RPA Green Light Institute for Emergency Care, Royal Prince Alfred Hospital, Sydney, Australia

**Keywords:** Low back pain, Emergency Medical Services, Hospitals, Multilevel analysis

## Abstract

**Background:**

One in 6 patients with low back pain (LBP) presenting to emergency departments (EDs) are subsequently admitted to hospital each year, making LBP the ninth most common reason for hospital admission in Australia. No studies have investigated and quantified the extent of clinical variation in hospital admission following an ED presentation for LBP.

**Methods:**

We used routinely collected ED data from public hospitals within the state of New South Wales, Australia, to identify presentations of patients aged between 18 and 111 with a discharge diagnosis of LBP. We fitted a series of random effects multilevel logistic regression models adjusted by case-mix and hospital variables. The main outcome was the hospital-adjusted admission rate (HAAR). Data were presented as funnel plots with 95% and 99.8% confidence limits. Hospitals with a HAAR outside the 95% confidence limit were considered to have a HAAR significantly different to the state average.

**Results:**

We identified 176,729 LBP presentations across 177 public hospital EDs and 44,549 hospital admissions (25.2%). The mean (SD) age was 51.8 (19.5) and 52% were female. Hospital factors explained 10% of the variation (ICC = 0.10), and the median odds ratio (MOR) was 2.03. We identified marked variation across hospitals, with HAAR ranging from 6.9 to 65.9%. After adjusting for hospital variables, there was still marked variation between hospitals with similar characteristics.

**Conclusion:**

We found substantial variation in hospital admissions following a presentation to the ED due to LBP even after controlling by case-mix and hospital characteristics. Given the substantial costs associated with these admissions, our findings indicate the need to investigate sources of variation and to determine instances where the observed variation is warranted or unwarranted.

**Supplementary information:**

The online version contains supplementary material available at 10.1186/s12913-022-08134-8.

## Background

Low back pain (LBP) is a leading condition for presentation to EDs, accounting for 4.4% of all presentations worldwide [1]. In Australia, LBP is the fifth most common reason for ED presentations [2]. Not only is LBP common in that setting, but there is evidence that the demand for ED services because of LBP has been increasing over time at a rate above that explained by population growth [3].

Despite most LBP presentations being classified as semi-urgent or non-urgent [3, 4], patients seeking emergency care for LBP typically have higher pain and disability scores [5]. They are also associated with substantial costs. For example, in Australia the average cost of an episode of care in the ED is $2,959. Costs are over four times higher when patients are subsequently admitted to hospital wards [6].

About 1 in 6 patients presenting to an ED in Australia with LBP are admitted to hospital [7] making LBP the ninth most common reason for hospital admission [2]. This figure is substantially higher than that reported in studies conducted in other countries such as The United States [8] and Canada, where the percentage of admitted patients appears to be around 2 to 6% [9, 10].

With high numbers of hospital admissions for LBP in Australia, it is important to determine whether this is a widespread occurrence, or if there is variation across hospitals. Variation raises questions about the quality, equity, efficiency of resource allocation and use of services, has implications for health services planning and policy, and is an important step towards improving patient care [11, 12]. Variation has been extensively documented across a range of healthcare settings (e.g. primary care and hospital settings) and services (e.g. such as diagnosis, treatment and prescribing practices) [12, 13]. However, we are unaware of other study that has investigated variation in hospital admissions subsequently to an EDs for LBP.

To address this research gap, this study aimed at identified and quantified the extent of clinical variation in hospital admission following an ED presentation for LBP across public hospitals in New South Wales, Australia.

## Methods

### Design

This is a retrospective study using routinely collected data and reported per the RECORD Statement [14]. The University of Sydney Human Research Ethics Committee exempted the present study from ethics approval because data were de-identified, not easily re-identifiable, and were not part of a linkage study.

### Data sources and settings

We obtained data from the New South Wales Emergency Department Records for Epidemiology (EDRE), which is sourced from the routinely collected ED data used for population health purposes by the New South Wales Ministry of Health. EDRE contains de-identified administrative data and includes hospital-level as well as patient-level data detailing ED presentations at public hospitals in New South Wales, Australia. Each record in the database represents a presentation. As data are de-identified, each presentation is assumed to be independent. We obtained data for all presentations from 2016 to 2019 as coverage in these years was 100% across metropolitan and rural hospitals. There are no data from private emergency departments in this dataset.

### Study population

We included all ED presentations of patients aged between 18 and 111 (the age of the oldest person living in Australia at the time data were collected) with a discharge diagnosis of LBP with or without neurological signs and symptoms. Cases were identified using the International Classification of Diseases (ICD) ICD-10, ICD-9 and the Systematised Nomenclature of Medicine Clinical Terms (SNOMED) codes. Identification of relevant diagnostic codes for LBP was performed by two researchers independently as described elsewhere [3]. The list of included diagnostic codes is described in Supplementary file 1.

We excluded LBP presentations due to serious spinal pathology, such as fractures, tumours, infections, cauda equina syndrome, myelopathy, etc. This decision was made as the expected treatment pathway for patients diagnosed with a serious spinal pathology is more likely to be hospital admission. Presentations without a diagnostic code were also excluded.

### Data collection

We sourced case-mix and hospital variables from the EDRE database. Case-mix variables included demographic information (age, sex, and whether English was the patient’s preferred language at home), the referral source (self-referral, general practitioner, specialist, aged care facility or other), whether the presentation arrived at the ED by ambulance, the triage category assigned to them using the Australasian Triage Scale (ATS), whether the presentation had occurred after hours (i.e. between 5 pm and 07:59 am or during weekends but not including Public holidays), and the type of LBP (with or without neurological signs and symptoms).

Hospital-level variables included geographical location (metropolitan or rural and regional areas of New South Wales) and the peer-group they belonged to [15]. In Australia, hospitals are categorised according to shared characteristics such as patient volumes, range of services provided, and level of specialisation of service into peer-groups [16]. We collapsed peer-group categories into the following: Principal referral, major, district, community, multi-purpose, sub-acute, and ungrouped hospitals. Supplementary file 2 describes hospital peer-groups relevant to this study.

### Outcomes

Our main outcome was the hospital-adjusted admission rate (HAAR). We also investigated the magnitude of the contextual effect of hospital – that is, the contribution of a hospital environment on their patients’ likelihood of being admitted.

### Case-mix adjustment

Case-mix variables included in the model were sex, age (centred around its mean), language spoken at home (English or other), acuity (urgent vs semi-urgent), arrival mode (ambulance vs others), type of LBP (with or without neurological signs and symptoms), and time of presentation (working hours vs after hours). We dichotomised the acuity based upon the Australasian Triage Scale (ATS) as urgent (ATS 1–3) or semi-urgent (4–5) as semi-urgent presentations are often used as one criterion to classify presentations as low-acuity [17]. After hours presentations were defined as those occurring between 5 pm and 7:59am or during weekends. These variables were chosen based on available data suggesting that they are predictors of admission [7]. Hospital peer-group was entered as a hospital variable.

### Analyses

We fitted random effects multilevel logistic regression models using *melogit* in Stata 17 (StataCorp LLC, College Station, Texas, US), where the hospitals’ specific effects were modelled through a normally distributed random intercept. This type of model account for the potential correlation of patient outcomes clustered within hospitals by modelling the variation of patients’ outcomes within hospitals and between hospitals using a random effect. Modelling these variance components not only affords to reduce the extent of unexplained variation but is of interest in its own, allowing to predict the hospital-specific effect while borrowing strength from all patients across different hospitals [18].

We assessed the discriminative ability of the multilevel model with the c-statistic by comparing the area under the curve of a model with case-mix variables only and no random effects to the multilevel model described above. A value of 1 indicates that the model perfectly discriminates individuals who were admitted (versus not admitted) as a function of individuals’ predicted probabilities, whereas a value of 0.5 represents random discrimination [19, 20]. Values between 0.8 and 0.9 represent excellent discrimination [21]. We also assessed the goodness-of-fit of the multilevel model by inspecting plots of observed versus predicted hospital admission after dividing the data into 10 deciles on the basis of predicted probabilities (Supplementary file 3)  [20].

We quantified the contextual hospital effect with the Intraclass Correlation Coefficient (ICC) and the median odds ratio (MOR). The ICC measures the proportion of observed variation in admission rates that is attributable to differences across hospitals (while accounting for case-mix). ICC values range from 0 to 1; an ICC = 0 indicates perfect independence of residuals; i.e., there is no influence of hospital on the outcome admission. An ICC = 1 means that the outcome (i.e. hospital admission) is entirely explained by which hospital that person presented to [19, 22]. The MOR can be conceptualised as the (median) increase in the odds of hospital admission if a patient with LBP went to a different hospital with higher odds of admitting patients [19, 23, 24].

We first fitted a model containing only case-mix variables (model 1) so that hospitals could be compared globally [25]. We subsequently fitted a model adjusted for both case-mix and hospital peer-group (model 2). For each model, we computed the HAAR for each hospital by dividing the predicted (P) by expected (E) probability of hospital admission within each hospital. The P/E ratio was then multiplied by the state average hospital admission rate. The P/E ratio is a modification of the commonly used observed to expected ratio, as the predicted probability of admissions (P) includes both the average intercept and the hospital-specific random effects [26]. This approach shrinks estimates towards the mean producing more precise estimates and is more robust to small sample sizes as it reduces the likelihood of classifying a hospital with a small volume as having larger or lower odds of hospital admission than an average hospital [20, 27].

HAAR values were plotted onto a ‘control chart’, or funnel plot. Funnel plots are preferable to other methods used to compare institutional performance [28, 29] and prompt more appropriate actions from decision-makers compared to other methods such as league tables [30]. We plotted HAARs against the number of LBP presentations. We used 95 and 99.8% confidence limits below and above the state average to characterise hospitals with low and higher admission rates [31]. Hospitals with a HAAR within the 95% confidence limits were classified as having an admission rate comparable to the state rate.

## Results

### Sample characteristics

From January 2016 to December 2019, there were 11,516,331 presentations to EDs in public hospitals across New South Wales. After excluding ineligible and non-LBP presentations, 176,729 were retained in the analysis. Characteristics of the included presentations are presented in Table 1. The mean (SD) age of the entire sample was 51.8, 28.4% were aged 65 and older, and 52% were female. Most presentations were classified as semi-urgent or urgent, and 92.5% of them were for LBP without neurological signs and symptoms. “Backache” was the most common diagnostic code (*n* = 72,199; 40.9%), followed by “low back pain” (*n* = 70,365; 39.8%), and sciatica (*n* = 9,738; 5.5%).

**Table 1:** Characteristics of presentations

	Total (*n* = 176,729)	Non-admitted (*n* = 132,270)	Admitted (*n* = 44,459)
Age, mean (SD)	51.8 (19.5)	48.4 (18.2)	61.8 (19.9)
Age 65 + , n (%)	50,269 (28.4)	28,141 (21.2)	22,128 (49.7)
Sex, n (%)^a^			
Female	91,906 (52)	66,868 (50.6)	25,038 (56.3)
Male	84,818 (48)	65,398 (49.4)	19,420 (43.7)
English as preferred language, n (%)	158,489 (89.7)	119,836 (90.6)	38,653 (86.9)
Referral source, n (%)^b^			
Self-referral	163,597 (92.6)	124,008 (93.8)	39,589 (89.1)
General practitioner	5,656 (3.2)	3,793 (2.9)	1,863 (4.2)
Aged care facility	1,100 (0.6)	438 (0.3)	662 (1.5)
Specialist services	326 (0.2)	153 (0.1)	173 (0.4)
Other	6,016 (3.4)	3,853 (2.9)	2,163 (4.8)
Arrival at ED by ambulance, n (%)^c^	55,788 (31.6)	28,744 (21.7)	27,044 (61)
Triage category (ATS), n (%)^d^			
1 (Resuscitation)	70 (0.04)	23 (0.02)	47 (0.1)
2 (Emergency)	5,631 (3.2)	3,329 (2.5)	2,302 (3.2)
3 (Urgent)	58,305 (33)	38,279 (28.9)	20,026 (33)
4 (Semi-urgent)	101,279 (57.3)	80,067 (60.5)	21,212 (57.3)
5 (Non-urgent)	11,417 (6.4)	10,547 (8)	870 (6.4)
Type of low back pain, n (%)			
Non-specific	163,543 (92.5)	121,838 (92.1)	41,705 (93.8)
Radicular	13,186 (7.5)	10,432 (7.9)	2,754 (6.2)
Hospital location			
Metropolitan Sydney Area	91,024 (53.2)	64,467 (48.7)	29,557 (66.5)
Rural or Regional NSW	82,705 (46.8)	67,803 (51.3)	14,902 (33.5)

^a^5 missing values (0.002%)

^b^34 missing values (0.01%)

^c^114 missing values (0.06%)

^d^27 missing values (0.015%)

Presentations occurred at 177 public hospital EDs across New South Wales. Of these, 142 were in rural or regional areas of New South Wales, and 35 in the metropolitan Sydney area. Thirteen EDs were in principal referral hospitals, 21 in major hospitals, 40 in district hospitals, 34 in community hospitals, 60 in multi-purpose hospitals, 2 in sub-acute hospitals, and 7 in ungrouped hospitals. Principal referral, major, and district hospitals were responsible for most presentations (*n* = 156,285, 88.4%). Across all seven peer-groups, presentations were similar in terms of age, percentage of presentations aged 65 and older. Principal referral hospitals had a higher proportion of presentations arriving by ambulance compared to major, district, community, multi-purpose, and sub-acute hospitals. They also had a lower proportion of presentations classified as non-urgent and higher proportion of presentations classified as urgent compared to all other peer-groups (Supplementary file 4).

There were 44,459 hospital admissions due to LBP from 2016–2019, representing an unadjusted hospital admission rate of 25.2%. Principal referral hospitals had the highest absolute number and proportion of patients admitted (*n* = 20,078, 35.5%), followed by sub-acute (33.9%), major (26.2%), ungrouped (21.6%), multi-purpose (17.4%), community (17%), and district (13.5%) hospitals. Within hospitals, the proportion of presentations that were admitted ranged from 0% (11 hospitals) to 46.8% (1 hospital).

### Model fit and discrimination

The adjusted models (c-statistic = 0.824 for both) were better at correctly discriminating presentations that resulted in hospital admission compared to the fixed effects-only logistic regression model (0.781). Likewise, the adjusted models fitted the data well (Supplementary file 3). Both models are described in Table 2.

**Table 2:** Parameter estimates, ICC, and MOR for models 1 and 2

Case-mix variabless	OR (95% CI)	
	**Model 1** **(Case-mix only)**	**Model 2** **(Case-mix + hospital)**
Female sex	1.16 (1.13 to 1.19)	1.16 (1.13 to 1.19)
Age^a^	1.03 (1.03 to 1.03)	1.03 (1.03 to 1.03)
English as primary language	1.07 (1.01 to 1.12)	1.07 (1.02 to 1.12)
Emergency department triage (“Urgent” or worse)	2.25 (2.12 to 2.40)	2.25 (2.12 to 2.40)
LBP without neurological signs and symptoms	1.23 (1.13 to 1.35)	1.23 (1.13 to 1.35)
Arrival at ED via ambulance	4.37 (3.95 to 4.83)	4.37 (3.94 to 4.83)
Presentation during working hours	1.14 (1.09 to 1.19)	1.14 (1.09 to 1.19)
Hospital peer-group		
Principal referral (ref)	-	-
Major	-	0.58 (0.39 to 0.86)
District	-	0.25 (0.20 to 0.32)
Community	-	0.40 (0.30 to 0.52)
Multi-purpose	-	0.35 (0.26 to 0.46)
Sub-acute	-	0.35 (0.49 to 2.57)
Ungrouped	-	0.19 (0.08 to 0.46)
Intercept	0.05	0.12
Variance of random intercept	0.55	0.39
ICC (95% CI)	0.14 (0.12 to 0.17)	0.10 (0.08 to 0.13)
MOR	2.03	1.81

*ICC* Intraclass correlation coefficient, *MOR* Median Odds Ratio, *LBP* Low back pain

^a^Age was centred around its mean

### Contextual effects

The ICC (95% CI) adjusted only for case-mix was 0.14 (0.12 to 0.17) and 0.10 (0.08 to 0.13) when also adjusted for hospital peer-group. This means that 14% of the variation observed in the model adjusted for case-mix only was attributed to differences across hospitals. This variation reduced to 10% when the model was adjusted for hospital peer-group. The MOR for the case-mix adjusted model was 2.03. After adjusting for hospital peer-group, the MOR reduced to 1.8.

### Hospital-adjusted admission rates

**Fig. 1:**
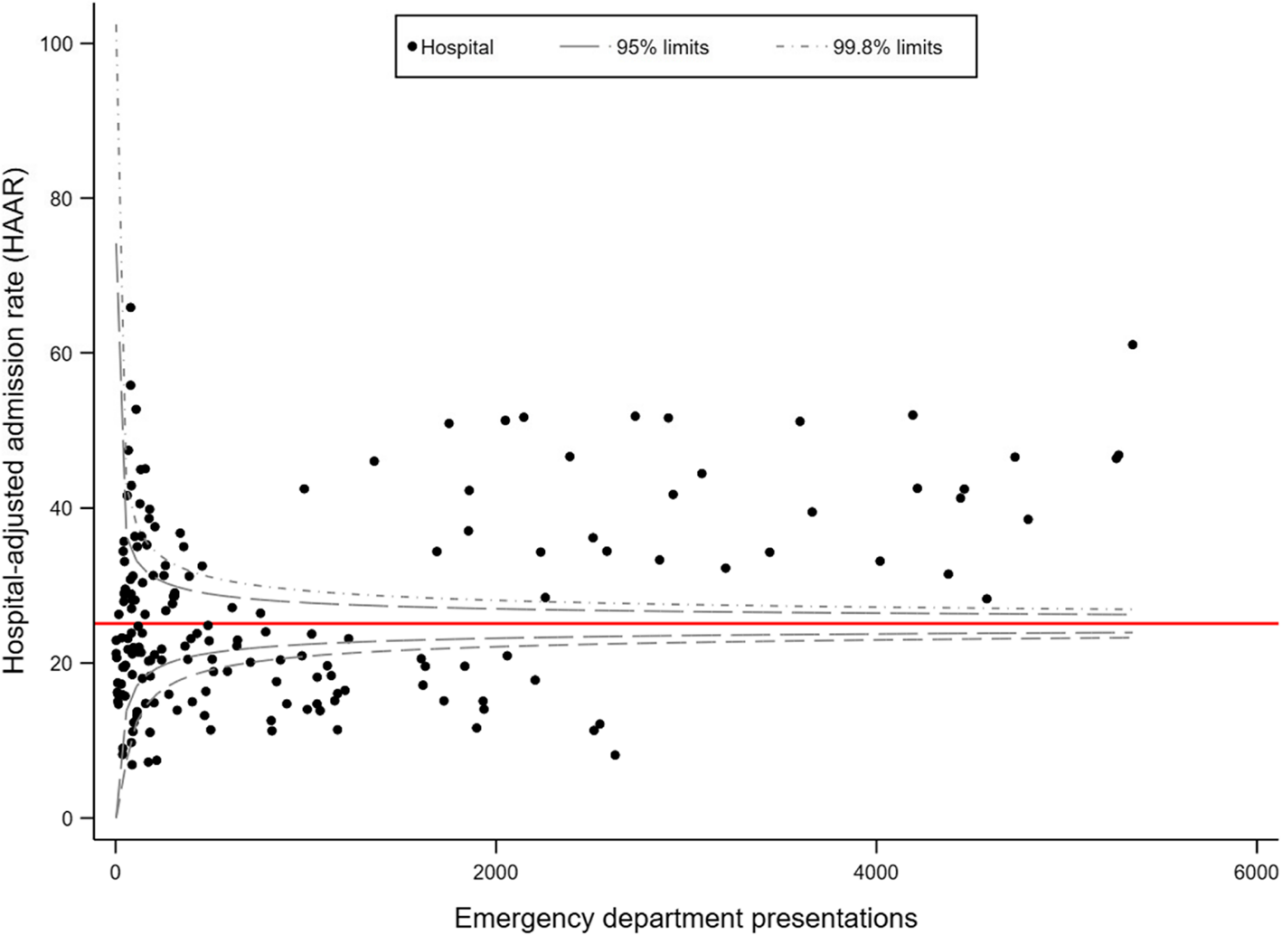
Funnel plot displaying the hospital-adjusted admission rate (HAAR) for 177 public hospital emergency departments in New South Wales using data from model 1 (adjusted for case-mix only). The red reference line is drawn at the average state admission rate of 25.2%

**Fig. 2:**
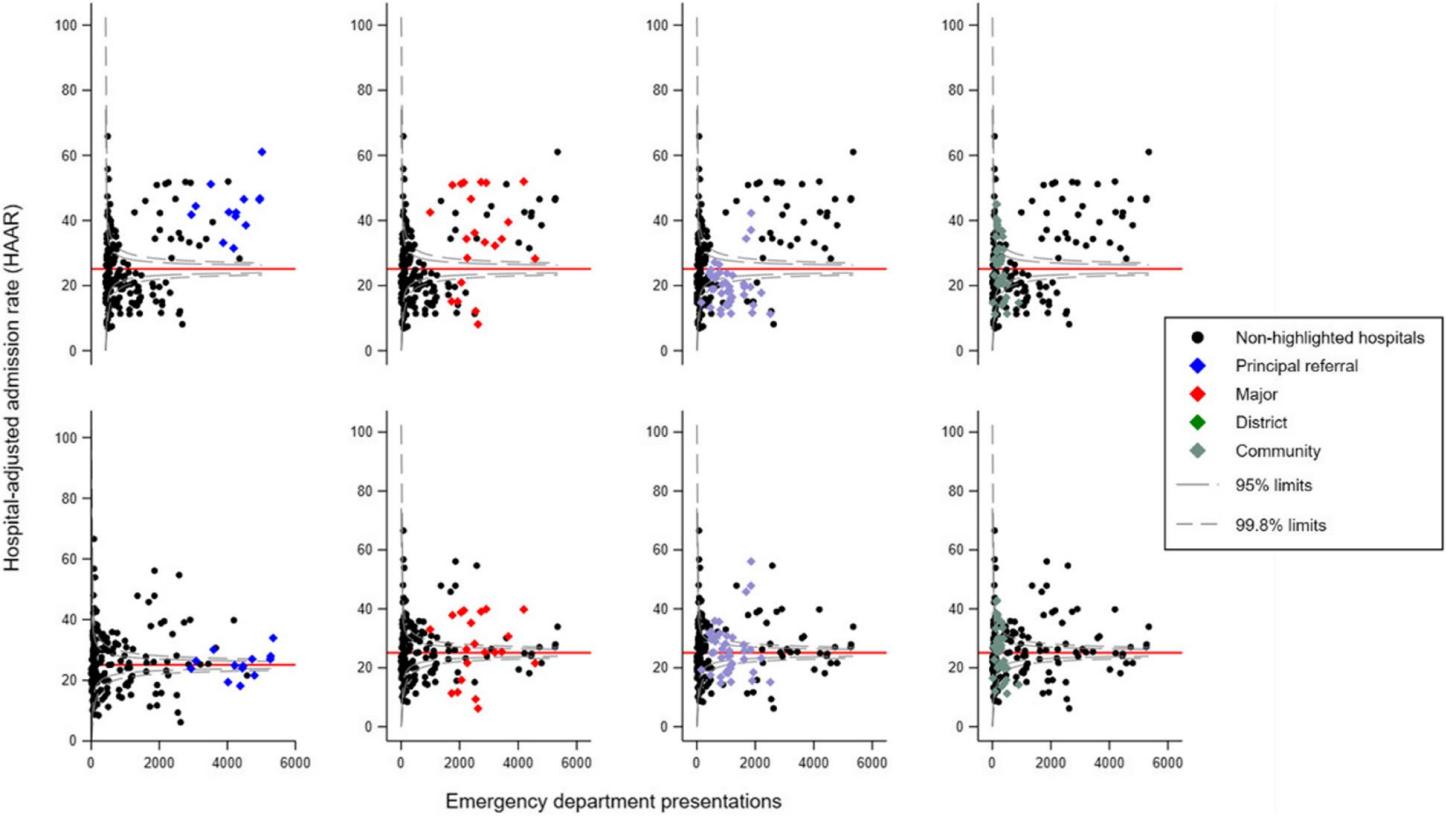
Funnel plots displaying hospital-adjusted admission rates (HAAR) estimated in model 1 (top row) and model 2 (bottom row). Coloured symbols highlight four distinct hospital peer-groups. Black dots represent non-highlighted hospitals pertaining to all other peer groups. The red reference line is drawn at the average state admission rate of 25.2%. Hospitals in the multipurpose and ‘other’ peer groups were omitted from this figure

There was marked variation in the HAAR across hospitals in both models. In model 1, HAAR ranged from 6.9 to 65.9%. This variation was observed in hospitals with both small and large numbers of LBP presentations (Fig. 1). Upon stratifying results in model 1 (Fig. 2, top row), larger hospitals had a higher proportion of hospitals classified as having a high admission rate. For example, all principal referral hospitals and 76.2% of major hospitals fell within that category compared to 7.5%, 35.3%, 18.8%, and 28.5% of district, community, multipurpose, and other hospitals, respectively. There were also hospitals that had a lower HAAR in relation to the state average, with 23.8% of major, 55% of district, 14.7% of community, 6.6% of multi-purpose, and 14.3% of other hospitals being classified as such.

Model 2, which adjusted the multilevel model for peer-group characteristics, provided distinct results. Less principal referral hospitals were classified as having high HAARs; 38.5% were classified as having admission rates comparable to the state average, and 23.1% as having admission rates lower than the stage average. Similar findings were noted for major hospitals, whereas more district hospitals had a HAAR within the 95% confidence limits (Fig. 2, bottom row).

## Discussion

### Summary of main findings

This study estimated the hospital admission rates in a large administrative dataset of people with LBP presenting to EDs across 177 public hospitals and found substantial variation in admission rates after controlling for case-mix and hospital variables. Our global comparison identified many hospitals that had HAAR higher and lower than the state average. After adjusting for hospital peer-group (model 2), we also identified substantial variation within each of these peer-groups – although less pronounced than in model 1. Contextual factors explained 10% to 14% of the variation observed in the multilevel models. We have also shown that the MOR ranged from 1.8 to 2. This means that if two patients with LBP and similar characteristics presented to hospitals with different admission rates, the patient presenting to the hospital with the higher admission rate was twice as likely to be admitted.

### Strengths and weaknesses of the study

Our study is the first to investigate hospital variation in admissions subsequently to an ED visit because of an episode of LBP adjusting for case-mix variables. Our models included known strong predictors of hospital admission (e.g. arriving by ambulance) and modelled data using a multilevel structure with a random effect for hospital which recognises the multilevel nature of our data [19]. Furthermore, each hospital-specific random effect was modelled using an approach that shrinks estimates towards the mean, which produces more precise estimates and is robust to small sample sizes as it reduces the likelihood of classifying a hospital with a small volume as having exceptional or poor results [20, 27].

Variation in the provision of health services has traditionally been measured in terms of geographical variation [32]. An example is the Australian Atlas of Healthcare Variation, which highlights geographical areas where use of certain services is higher or lower than the national average [32]. The approach used in our study focused on describing variation across hospitals, which has been shown to be a more important factor to determine the type of treatment a patient receives than the area in which they live [33].

To the best of our knowledge, there are no validated risk adjustment tools for hospital admission following an ED presentation in people with low back pain. This means that for this study we had to rely on factors identified as predictive of hospital admission in previous studies [7]. We also used administrative ED data, and thus some potentially important key case-mix variables, such as the severity of LBP (e.g. pain intensity), were not present in our models and could be considered a limitation of this study. Nevertheless, both adjusted models had excellent discriminatory ability (c statistic = 0.824). One explanation is that the severity of LBP might is indirectly captured by other variables available on our case-mix adjusted models. For example, pain intensity is a factor to be considered by triage nurses when assigning a patient to a certain triage category. The Australasian College for Emergency Medicine guidelines for the implementation of the ATS provide guidance on how to triage patients based upon pain intensity (e.g. pain that is very severe should be classified as ATS 2; minimal pain should be classified as ATS 5) [34].

Compared to fixed-effects models, random effects models as employed by our study typically provide results that are more specific than sensitive. As a consequence, our analytical approach can be considered more conservative as it may have classified some hospitals that are true outliers as being no different than the average [18]. Given the current uncertainty around which admissions due to LBP are warranted or unwarranted, we believed that it was more appropriate to adopt a more conservative approach.

### Meaning of the study and future directions

As LBP is the fifth most common reason why people in Australia present to EDs and the ninth most common reason for hospital admission following an ED presentation, quantifying variation in admission rates across hospitals in the most populous state of Australia is an important step towards hospital care for LBP. By identifying substantial variation across hospitals with distinct characteristics and those that share similar characteristics (i.e., within the same peer-group), the next logical step is to determine instances where the observed variation is warranted or unwarranted. However, there is currently no consensus on the criteria to define a hospital admission for LBP as either warranted or unwarranted. Existing tools commonly used to determine which ED presentations are low acuity (and thus potentially unwarranted) are unlikely to be informative as patient admission is often used as a criteria to automatically classify a presentation as warranted [35].

Variation across hospitals as observed in our study is common but is not ubiquitous. In a similar subset of hospitals as that described in our study, Falster et al. [36] showed that there is minimal variation in admissions across hospitals for conditions such as acute myocardial infarction and hip fractures, although substantial variation was found for preventable hospitalisations (e.g. asthma, heart failure, hypertension, etc.). It is difficult to ascertain causes of variation in the context of their and our analysis given their observational nature, however the existence of more concrete guidance on the available clinical pathways, including hospital admission, for patients experiencing a myocardial infarction or a hip fracture in Australia, might help explain the distinct findings [37, 38]. There is very little guidance on how to manage LBP in the ED and what constitutes an appropriate hospital admission for LBP. Most evidence that informs the management of LBP in that setting is from studies conducted in primary care and is mostly silent on hospital admissions  [39].

Two unmeasured factors in our study that warrant further investigation are the treating clinician and patients’ social factors. Clinicians are ultimately responsible for deciding whether to admit a patient, although hospital factors such as culture and policies might shape clinicians’ behaviour. Social factors, lack of outpatient social support, were also not present in our model, but seem to be an important contributing factor to emergency doctors’ decision to admit a patient [40]. This seems particularly relevant in the Australian context, as patients living in areas classified as being of low socioeconomic status make up more than half of non-urgent triage category presentations in EDs [2].

## Conclusions

We found substantial variation in hospital admissions following a presentation to the ED due to LBP even after controlling by case-mix and hospital characteristics. Given the substantial costs associated with hospital admissions following an ED presentation for LBP, our findings indicate the need to investigate sources of variation and to determine instances where the observed variation is warranted or unwarranted.

## Supplementary information

**Additional file 1.**

## Data Availability

The data used in this study is under the custody of New South Wales Health and requires their approval to be accessed.
