# Supplementary material for: Hospital variation in admissions for low back pain following an emergency department presentation: a retrospective study
Source: BMC Health Serv Res. 2022 Jul 12;22:835. doi: 10.1186/s12913-022-08134-8 (PMC9275239; doi:10.1186/s12913-022-08134-8)
Supplement: Supplementary file 1 — Additional file 1. [file 12913_2022_8134_MOESM1_ESM.docx]

**Supplementary file 1.** Diagnostic codes for low back pain and low back pain with neurological signs and symptoms

| Diagnostic system | Diagnostic code | Frequency |
| --- | --- | --- |
| **Low back pain** | | |
| SNOMED | Backache (finding) | 72199 |
| SNOMED | Low back pain (finding) | 48212 |
| ICD | M54.5 - Low back pain (Ed.1-Ed.9) | 22153 |
| ICD | M54.99 - Unspecified dorsalgia, site unspecified (Ed.1-Ed.9) | 5919 |
| SNOMED | Acute low back pain (finding) | 1582 |
| ICD | 724.5 - Other and unspecified disorders of back, Backache, unspecified | 1540 |
| SNOMED | Lower back injury (disorder) | 1403 |
| SNOMED | Lumbar sprain (disorder) | 1237 |
| SNOMED | Strain of back muscle (disorder) | 1137 |
| SNOMED | Sacral back pain (finding) | 996 |
| SNOMED | Spasm of back muscles (finding) | 971 |
| ICD | 724.2 - Other and unspecified disorders of back, Low back pain | 531 |
| ICD | S33.7 - Sprain and strain of other and unspecified parts of lumbar spine and pelvis (Ed.1-Ed.9) | 520 |
| SNOMED | Sacroiliac joint pain (finding) | 502 |
| SNOMED | Chronic low back pain (finding) | 481 |
| SNOMED | Pain in the coccyx (finding) | 457 |
| SNOMED | Injury of coccyx (disorder) | 433 |
| SNOMED | Injury of back (disorder) | 405 |
| SNOMED | Back pain complicating pregnancy (disorder) | 354 |
| SNOMED | Mechanical low back pain (finding) | 345 |
| SNOMED | Chronic back pain (finding) | 279 |
| SNOMED | Sprain of sacroiliac ligament (disorder) | 227 |
| ICD | M53.80 - Other specified dorsopathies, multiple sites in spine (Ed.1-Ed.9) | 159 |
| SNOMED | Lumbar spondylosis (disorder) | 139 |
| SNOMED | Complaining of low back pain (finding) | 132 |
| SNOMED | Degeneration of lumbar intervertebral disc (disorder) | 122 |
| SNOMED | Spondylosis (disorder) | 113 |
| ICD | M51.2 - Other specified intervertebral disc displacement (Ed.1-Ed.9) | 107 |
| SNOMED | Coccyx sprain (disorder) | 93 |
| SNOMED | Sacrum sprain (disorder) | 92 |
| SNOMED | Low back strain (disorder) | 88 |
| ICD | M53.3 - Sacrococcygeal disorders, not elsewhere classified (Ed.1-Ed.9) | 88 |
| ICD | 724.8 - Other and unspecified disorders of back, Other symptoms referable to back | 62 |
| SNOMED | Sprain of ligament of lumbosacral joint (disorder) | 61 |
| ICD | S33.50 - Sprain and strain of lumbar spine, unspecified (Ed.1) | 46 |
| ICD | M47.99 - Unspecified spondylosis, site unspecified (Ed.1-Ed.9) | 45 |
| SNOMED | Superficial injury of back (disorder) | 40 |
| SNOMED | Displacement of lumbar intervertebral disc without myelopathy (disorder) | 37 |
| SNOMED | Injury of muscle and tendon of abdomen, lower back and pelvis (disorder) | 31 |
| ICD | 722.10 - Intervertebral disc disorders, Displacement of thoracic or lumbar intervertebral disc without myelopathy, Lumbar intervertebral disc without myelopathy | 25 |
| ICD | 724.79 - Other and unspecified disorders of back, Disorders of coccyx, Disorders of coccyx, other | 25 |
| SNOMED | Exacerbation of backache (finding) | 24 |
| SNOMED | Stiff back (finding) | 24 |
| ICD | M51.3 - Other specified intervertebral disc degeneration (Ed.1-Ed.9) | 23 |
| ICD | 724.6 - Other and unspecified disorders of back, Disorders of sacrum | 17 |
| ICD | 847.2 - Sprains and strains of other and unspecified parts of back, Lumbar | 15 |
| ICD | M51.8 - Other specified intervertebral disc disorders (Ed.1-Ed.9) | 10 |
| SNOMED | Sacral edema (disorder) | 7 |
| ICD | 722.52 - Intervertebral disc disorders, Degeneration of thoracic or lumbar intervertebral disc, Degeneration of thoracic or lumbar intervertebral disc, lumbar or lumbosacral intervertebral disc | 6 |
| SNOMED | Synovial cyst of sacrum (disorder) | 6 |
| ICD | 722.90 - Intervertebral disc disorders, Other and unspecified disc disorder, Other and unspecified disc disorder, unspecified region | 4 |
| SNOMED | Sprain of sacrococcygeal ligament (disorder) | 4 |
| ICD | 847.4 - Sprains and strains of other and unspecified parts of back, Coccyx | 3 |
| ICD | 722.2 - Intervertebral disc disorders, Displacement of intervertebral disc, site unspecified, without myelopathy | 2 |
| ICD | 847.3 - Sprains and strains of other and unspecified parts of back, Sacrum | 2 |
| ICD | 722.6 - Intervertebral disc disorders, Degeneration of intervertebral disc, site unspecified | 1 |
| SNOMED | Back pain prevention education (regime/therapy) | 1 |
| SNOMED | Entire sacroiliac joint (body structure) | 1 |
| SNOMED | Lumbar facet joint pain (finding) | 1 |
| SNOMED | Lumbar spine painful on movement (finding) | 1 |
| SNOMED | Lumbar spine stiff (finding) | 1 |
| SNOMED | Pain in lumbar spine (finding) | 1 |
| SNOMED | Pain radiating to lumbar region of back (finding) | 1 |
| **Low back pain with neurological signs and symptoms** | | |
| SNOMED | Sciatica (disorder) | 9738 |
| ICD | M54.3 - Sciatica (Ed.1-Ed.9) | 1436 |
| SNOMED | Lumbago with sciatica (finding) | 755 |
| SNOMED | Acute back pain with sciatica (finding) | 260 |
| SNOMED | Acute sciatica (disorder) | 218 |
| SNOMED | Spinal stenosis of lumbar region (disorder) | 212 |
| SNOMED | Lumbago-sciatica due to displacement of lumbar intervertebral disc (disorder) | 173 |
| SNOMED | Injury of lumbar nerve roots (disorder) | 132 |
| SNOMED | Lumbar radiculopathy (disorder) | 110 |
| SNOMED | Lumbar disc prolapse with radiculopathy (disorder) | 51 |
| SNOMED | Chronic sciatica (disorder) | 41 |
| SNOMED | Injury of sciatic nerve (disorder) | 18 |
| SNOMED | Injury of nerves and lumbar spinal cord at abdomen, lower back and pelvis level (disorder) | 14 |
| SNOMED | Injury of sacral nerve roots (disorder) | 12 |
| ICD | S74.0 - Injury of sciatic nerve at hip and thigh level (Ed.1-Ed.9) | 7 |
| ICD | S34.2 - Injury of nerve root of lumbar and sacral spine (Ed.1-Ed.9) | 2 |
| ICD | 953.5 - Injury to nerve roots and spinal plexus, Lumbosacral plexus | 1 |
| ICD | 956.0 - Injury to peripheral nerve(s) of pelvic girdle and lower limb, Sciatic nerve | 1 |
| SNOMED | Compression of sacral nerve root (disorder) | 1 |
| SNOMED | Injury of lumbosacral plexus (disorder) | 1 |
| SNOMED | Prolapsed lumbar intervertebral disc with sciatica (disorder) | 1 |
| ICD | S34.5 - Injury of lumbar, sacral and pelvic sympathetic nerves (Ed.1-Ed.9) | 1 |
| ICD | S346 - Injury of peripheral nerve(s) of abdomen, lower back and pelvis (Ed.1-Ed.9) | 1 |

ICD, International Classification of Diseases; SNOMED, Systematised Nomenclature of Medicine Clinical Terms

**Supplementary file 2. Hospital peer groups**

| **AIHW Criteria** | **Labels used in this study** |
| --- | --- |
| **Principal referral**  Greater than 35,000 acute weighted separations AND offering highly specialised services (such as bone marrow and other specialised transplants, severe burn injury, major trauma) | Principal referral |
| **Major hospitals group 1**  35,000 or less but greater than 17,000 acute weighted separations AND availability of one or more specialist services requiring specific infrastructure (such as cardiac catheterisation, comprehensive cancer centre, in-centre dialysis and medical radiation imaging) OR average acute NWAU per separation of 1.25 or greater | Major |
| **Major hospitals group 2**  35,000 or less but greater than 10,000 acute weighted separations AND no specialist services requiring specific infrastructure OR average acute NWAU per separation less than 1.25 |  |
| **District group 1**  10,000 or less but greater than 4,000 acute weighted separations | District |
| **District group 2**  4,000 or less acute weighted separations but greater than 2,000 acute separations |  |
| **Community with surgery**  2,000 or less acute separations but greater than 200 total separations AND more than 2 per cent surgery (based on DRG status) | Community |
| **Community without surgery**  2,000 or less acute separations AND less than 2 per cent surgery (based on DRG status), OR Less than 200 total separations |  |
| **Multi-purpose service**  Multi-purpose service, including associated aged care entity | Multipurpose |
| **Sub-acute**  75 per cent or more general (mixed) sub- and nonacute Activity | Other |
| **Other ungrouped**  Other sub- and non-acute not able to be grouped to any other category |  |
| **Ungrouped acute – tertiary referral**  Specialist acute role not suitable for any other rolebased group and tertiary referral status |  |

AIHW, Australian Institute of Health and Welfare; NWAU, National Weighted Activity Unit; DRG, Diagnosis-related group

**Supplementary file 3. Multilevel model discrimination and goodness-of-fit**

**C-statistic: discrmination in relation to a fixed-effects model only**

| **Model** | **Area under the curve (95% CI)** |
| --- | --- |
| Fixed effects only | 0.781 (0.778 to 0.783) |
| Random effects (case-mix only) | 0.824 (0.821 to 0.806) |
| Random effects (case-mix + hospital) | 0.824 (0.821 to 0.826) |

**Goodness-of-fit of random effects models. Predicted versus observed number of admissions across deciles for model A.**


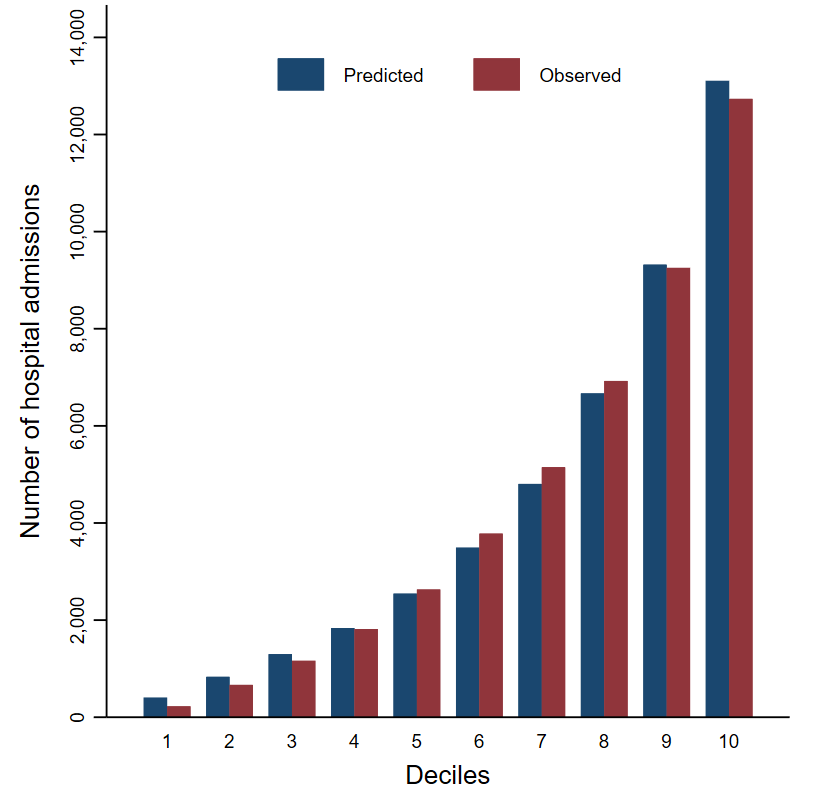


**Supplementary file 4. Characteristics of presentations stratified by hospital peer-group**

|  | **Principal referral**  **(n = 13)** | **Major**  **(n = 21)** | **District**  **(n = 40)** | **Community**  **(n = 34)** | **Multi-purpose**  **(n = 60)** | **Sub-acute**  **(n = 2)** | **Ungrouped**  **(n = 7)** |
| --- | --- | --- | --- | --- | --- | --- | --- |
| Number of presentations | 56,533 | 54,798 | 44,954 | 8,977 | 6,521 | 1,532 | 3,414 |
| Age, mean (SD) | 52 (19.9) | 51.8 (19.7) | 51.3 (19.1) | 52 (19.1) | 52.7 (18.8) | 55.8 (20.7) | 51.1 (19.1) |
| Age 65+, n (%) | 16,654 (29.4) | 15.789 (28.8) | 11,947 (26.58) | 2.539 (28.3) | 1.865 (28.6) | 555 (36.2) | 920 (26.9) |
| Sex, n (%)^a^ |  |  |  |  |  |  |  |
| Female | 29,141 (51.5) | 28,774 (52.5) | 23,189 (51.6) | 4,918 (54.8) | 3,403 (52.2) | 784 (51.2) | 1,697 (49.7) |
| Male | 27,389 (48.5) | 26,024 (47.5) | 21,765 (48.4) | 4,059 (45.2) | 3.118 (47.8) | 748 (48.8) | 1,715 (50.3) |
| Arrival at ED by ambulance, n (%)^c^ | 20,207 (35.8) | 18,570 (33.9) | 11,751 (26.1) | 2,029 (22.6) | 1,416 (21.8) | 489 (31.9) | 1,326 (38.9) |
| Triage category (ATS), n (%)^d^ |  |  |  |  |  |  |  |
| 1 (Ressuciation) | 54 (0.1) | 7 (0.01) | 6 (0.01) | 2 (0.02) | 1 (0.02) | 0 | 0 |
| 2 (Emergency) | 1,896 (3.3) | 1,900 (3.5) | 1,248 (2,78) | 302 (3.4) | 200 (3.1) | 39 (2.5) | 2 (1.3) |
| 3 (Urgent) | 22,230 (39.3) | 15,084 (27.5) | 14,650 (32.6) | 3,059 (34.1) | 2,358 (36.2) | 371 (24.2) | 553 (16.2) |
| 4 (Semi-urgent) | 30,327 (53.7) | 24,071 (62.2) | 25,966 (57.8) | 4,489 (50.1) | 2,894 (44.5) | 993 (64.82) | 2,539 (74.37) |
| 5 (Non-urgent) | 2,025 (3.6) | 3,735 (6.8) | 3,080 (6.9) | 1,117 (12.5) | 1,055 (16.2) | 129 (8.4) | 276 (8.1) |
| Type of low back pain, n (%) |  |  |  |  |  |  |  |
| Radicular | 4,041 (7.1) | 4,146 (7.6) | 3,782 (8.4) | 635 (7.1) | 288 (4.4) | 129 (8.4) | 165 (4.8) |
| Admitted to hospital, n (%) | 20,078 (35.5) | 14,377 (26.2) | 6,087 (13.5) | 1,528 (17) | 1,133 (17.4) | 520 (33.9) | 736 (21.6) |

ED, emergency department; ATS, Australasian Triage Scale. Percentages might not add up to 100 due to rounding
